# Proteomic Analysis of HDAC3 Selective Inhibitor in the Regulation of Inflammatory Response of Primary Microglia

**DOI:** 10.1155/2017/6237351

**Published:** 2017-02-15

**Authors:** Mingxu Xia, Qiuchen Zhao, He Zhang, Yanting Chen, Zengqiang Yuan, Yun Xu, Meijuan Zhang

**Affiliations:** ^1^Department of Neurology, Affiliated Drum Tower Hospital, Nanjing University Medical school, Nanjing, Jiangsu 210008, China; ^2^The State Key Laboratory of Brain and Cognitive Sciences, Institute of Biophysics, Chinese Academy of Sciences, Beijing 100101, China

## Abstract

HDAC3 has been shown to regulate inflammation. However, the role of HDAC3 in primary microglia is largely unknown. RGFP966 is a newly discovered selective HDAC3 inhibitor. In this study, we used protein mass spectrometry to analyze protein alterations in LPS-treated primary microglia with the application of RGFP966. Generally, about 2000 proteins were studied. 168 of 444 (37.8%) LPS-induced proteins were significantly reduced with the treatment of RGFP966, which mainly concentrated on Toll-like receptor signaling pathway. In this regard, we selected Toll-like receptor 2 (TLR2), TLR3, TLR6, MAPK p38, CD36, and spleen tyrosine kinase (SYK) for further validation and found that they were all significantly upregulated after LPS stimulation and downregulated in the presence of RGFP966. Additionally, RGFP966 inhibited supernatant tumor necrosis factor (TNF)-*α* and Interleukin 6 (IL-6) concentrations. Activation of STAT3 and STAT5 was partially blocked by RGFP966 at 2 h after LPS-stimulation. The fluorescence intensity of CD16/32 was significantly decreased in LPS + RGFP966-treated group. In conclusion, our data provided a hint that RGFP966 may be a potential therapeutic medication combating microglia activation and inflammatory response in central nervous system, which was probably related to its repressive impacts on TLR signaling pathways and STAT3/STAT5 pathways.

## 1. Introduction

Microglia are resident immune cells in the brain and play a pivotal role in immune surveillance. They are activated in diverse neurological diseases including encephalitis, stroke, Parkinson's disease, and Alzheimer's disease, resulting in the subsequent inflammatory cascade [1]. It is undisputable that inflammation is beneficial for homeostasis restoration and tissue repair by means of clearing pathogens and harmful cell components. However, excessive inflammation causes damage to brain tissues and exacerbates the initial insult. Therefore, the magnitude of microglia activation must be tightly controlled to avoid the collateral tissue damage and to regulate the progression of neurological diseases [2, 3].

Histone deacetylases (HDACs) are conserved metalloproteases which aim to remove acetyl groups from lysine residues of targeted proteins. In accordance with their structural diversity, HDACs are divided into four subtypes: Class I (HDAC1, HDAC2, HDAC3, and HDAC8), Class II consisting of IIa (HDAC4, HDAC5, HDAC7, and HDAC9) and IIb (HDAC6 and HDAC10), Class III (a family of sirtuins), and Class IV (HDAC11) [4]. Together with histone acetylases (HATs), HDACs regulate acetylation level of histones (H3, H2AK5, H4K5, H4K12, H2B, H4K8, and H4K16) as well as some other proteins (p65 and myocyte enhancer factor 2) [5]. Recently, several studies proposed that HDAC inhibitors are involved in modulating innate immune activity [6, 7] and could be potentially applied in various human diseases [8].

However, previous used broad-spectrum HDAC inhibitors target several HDACs and it is difficult to define exact role of each subtype. Additionally, clinical trials with pan-HDACi in cancer patients suffered undesired effects including increased susceptibility to pneumonia, thrombocytopenia, anorexia [9]. Thus, it is necessary to focus on the specific HDAC inhibitor.

HDAC3 is the most widely expressed HDACs in the brain [10] and is thought to play a role in Huntington [4], SCA [11], and dementia diseases [12]. HDAC3-deficient macrophages possessed decreased ability to activate inflammatory gene expression in response to LPS stimulation [13]. Concomitantly, HDAC3 was found to be an epigenomic brake in macrophage alternative activation [14], while inflammation repressive repertoire of HDAC3 in primary microglia is largely unknown. RGFP966 is a selective HDAC3 inhibitor, with an IC50 of 0.08 *μ*M and no effective inhibition of other HDACs at concentrations up to 15 *μ*M, and could cross brain blood barrier when administrated peripherally [15]. Pharmacological inhibition of HDAC3 may bring more evidences and prosperities for clinical applications. This insight now gives us the opportunity to study possible inflammatory consequences of HDAC3 in central nervous system.

In this issue, we used protein mass spectrometry to profile global molecular alterations in primary microglia exposed to RGFP966, exploring a potential signaling pathway through which HDAC3 specific inhibitor RGFP966 regulated inflammation.

## 2. Materials and Methods

### 2.1. Primary Microglia Culture and Treatment

Primary microglia cells were prepared from C57BL/6 mice born within 24 hours as previously described. Briefly, cerebral cortex tissue was digested in TrypLE for 10 minutes at 37°C. Then, Minimum Essential Medium (MEM) (Hyclone, USA) supplemented with 10% fetal bovine serum (FBS) (Biological Industries, Israel), 100 U/mL penicillin, and 100 ug/mL streptomycin was used to terminate digestion. Afterwards, the cells were centrifuged at 1500 rpm for 5 minutes, resuspended, and seeded in the 75 cm^2^ flasks. After 36–48 hours, the culture media were replaced by Dulbecco's Modified Eagle Media (DMEM) (Hyclone, USA) with 10% FBS, 100 U/mL penicillin, and 100 ug/mL streptomycin. At day 11–13, microglia cells were suspended and obtained by shaking the flasks at 180 rpm for 10 min at 37°C. The mature microglia cells were seeded into plates at a density of 2 × 10^5^/cm^2^ and placed for 36–48 hours before further treatment. RGFP966 (Selleckchem) was dissolved in dimethyl sulfoxide (DMSO) to make 55 mM stock solution. In purified enzyme assays, inhibition IC50 values of RGFP966 for HDAC1, HDAC2, and HDAC3 were >15 *μ*M, >15 *μ*M, and 0.08 *μ*M [15, 16]. Primary microglia cells were pretreated with DMSO or RGFP966 at a concentration of 15 *μ*M for an hour. Then, LPS (Sigma-Aldrich, USA) was added to the culture media at a dose of 500 ng/mL. Proteins, mRNA, and supernatant were collected for proteomic analysis, western blotting, CBA, and Q-PCR at indicated time points.

### 2.2. Proteomic Analysis

Proteomic analysis was performed by AB SCIEX TripleTOF 5600 mass spectrometer (AB SCIEX, USA) equipped with a liquid chromatography-tandem mass spectrometry (LC-MS/MS) system. Proteins (200 *μ*g) of four samples were resolved on 10% SDS polyacrylamide gels. The gels were stained with Coomassie Blue G-250 for 1 hour and then cut into blocks after distaining in ultrapure H_2_O. The gel blocks were digested using trypsin and peptides were extracted from them. After being dried and redissolved, the peptides were analyzed. The mass spectra were annotated against the Uniprot proteome database. By means of the Software DAVID coupled with STRING (version 10.0), the Gene Ontology analysis, KEGG pathway analysis, and protein-protein interactions were completed.

### 2.3. Real-Time PCR

As described previously, Trizol reagent (Invitrogen, USA) was used for the total RNA extraction from microglia cells and then RNA was reverse-transcribed into cDNA with a PrimeScript RT reagent Kit (Takara, Dalian, China). The quantitative measurements were performed on an ABI 7500 PCR instrument (Applied Biosystems, USA) with a SYBR green Kit (Takara, Dalian, China). Relative gene expressions were normalized to glyceraldehyde-3-phosphate dehydrogenase (GAPDH) and mRNA expression levels were presented as fold changes versus DMSO group. The primers (Invitrogen) used are as follows:

TLR-2 primers:

  Forward: 5′-TCACATGGCAGAAGATGTGTC-3′
  Reverse: 5′-GGTGATGCAATTCGGATGCT-3′

TLR-3 primers:

  Forward: 5′-TGAGAAGAGCCACAGTGATAGA-3′
  Reverse: 5′-CTCTCCAGCAGAAGAGACACAA-3′

TLR6 primers:

  Forward: 5′-AATGGTACCGTCAGTGCTGGA-3′
  Reverse: 5′-CTTGGCTCATGTTGCAGAGG-3′

CD36 primers:

  Forward: 5′-TGAATGGTTGAGACCCCGTG-3′
  Reverse: 5′-TAGAACAGCTTGCTTGCCCA-3′

GAPDH primers:

  Forward: 5′-GCCAAGGCTGTGGGCAAGGT-3′
  Reverse: 5′-TCTCCAGGCGGCACGTCAGA-3′.

### 2.4. Western Blot

Western blot was performed as previously described. Equal amounts of proteins were separated by sodium dodecyl sulfate-PAGE electrophoresis and then blotted onto polyvinylidene fluoride membranes. After being blocked in 5% fat-free milk for 2 hours, membranes were incubated with primary antibodies against TLR2 (1 : 1000, Abcam, UK), TLR3 (1 : 1000, Abcam, UK), TLR6 (1 : 1000, Cell Signaling Technology, USA), MAPK p38 (1 : 500, Cell Signaling Technology, USA), phospho-p38 (1 : 500, Cell Signaling Technology, USA), CD36 (1 : 1000, Abcam, UK), SYK (1 : 1000, Abcam, UK), STAT3 (1 : 500, Cell Signaling Technology, USA), phospho-STAT3 (1 : 500, Cell Signaling Technology, USA), STAT5 (1 : 500, Cell Signaling Technology, USA), phospho-STAT5 (1 : 500, Cell Signaling Technology, USA), and GAPDH (1 : 5000, Bioworld, USA) in 4°C overnight to probe targeted proteins. Horseradish peroxidase-conjugated secondary antibodies (1 : 5000, Bioworld, USA) were used to combine primary antibodies and the reaction was detected with an ECL Kit (Bioworld, USA). The intensities of blots were quantified by densitometry.

### 2.5. Immunofluorescence

Primary microglia cells seeded on cover slips were fixed with 4% polyformaldehyde for 15 minutes and permeabilized with 0.2% Triton-X100 for 20 minutes at room temperature. After being blocked in 2% BSA in PBS for 2 hours, microglia cells were incubated with donkey anti-CD16/32 antibody (1 : 500, BD Pharmingen, USA) overnight in 4°C and subsequently incubated with FITC-conjugated anti-rat IgG (Invitrogen, USA) at room temperature for 2 hours. DAPI staining was used to localize the nuclei. Images were taken by a fluorescence microscope (Olympus, Japan) and fluorescence intensities were analyzed by ImageJ software (version 1.39, National Institutes of Health, USA).

### 2.6. Quantification of Secreted Cytokines

Cytokines in the supernatants were measured using the Cytometric Bead Array (CBA) Mouse Inflammation Kit (BD Biosciences, USA). Briefly, after incubation with capture beads on which anti-cytokine antibodies are coated and PE-conjugated anti-cytokine antibodies, cytokine levels can be quantitatively analyzed. The assays were performed by BD Accuri C6 flow cytometer (BD Biosciences, USA) following the manufacturer's instructions and the data were generated with FCAP Array version 3.0.1 Software.

### 2.7. Statistical Analysis

Data were expressed as mean ± SD of three independent experiments. Comparisons between groups were conducted with SPSS 22.0 software. Differences were analyzed by two-way analysis of variance (ANOVA) followed by Bonferroni's post hoc test and considered to be statistically significant if *p* < 0.05.

## 3. Results

### 3.1. Overview of Proteomic Analysis

**Figure 1:**
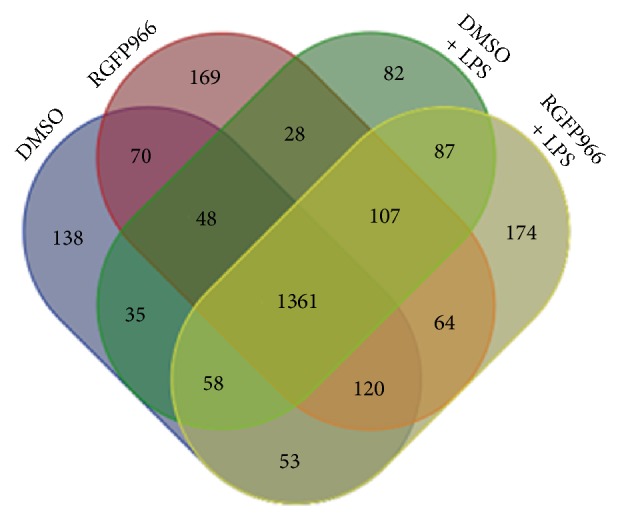
Overview of expressed proteins in four groups. The blue circle represented DMSO group, the red circle represented RGFP966 group, the green circle represented DMSO + LPS group, and the yellow circle represented RGFP966 + LPS group.

In this study, 1883 proteins were detected in DMSO group and 1967 proteins were in RGFP966 group. In LPS-stimulated groups, there were 1806 proteins in DMSO + LPS group and 2024 proteins in RGFP966 + LPS group (Table 1). A Venn diagram showed the relationship of expressed proteins in four groups (Figure 1).

### 3.2. Alterations in Proteins Expression

**Figure 2:**
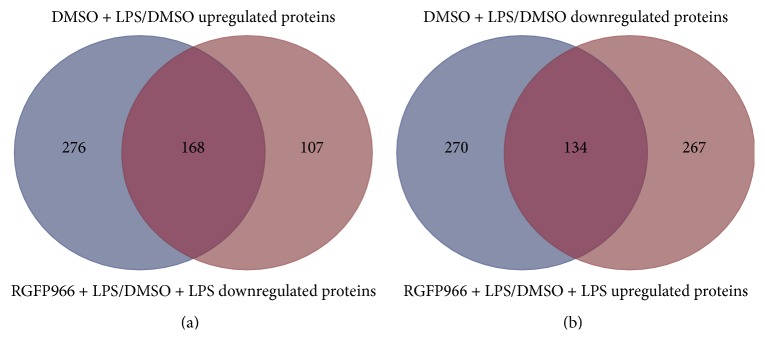
Venn diagrams of the differentially expressed proteins between LPS-stimulated (DMSO + LPS/DMSO) and RGFP966-treated (RGFP966 + LPS/DMSO + LPS) microglia cells. (a) 168 proteins were upregulated by LPS and reversed with the treatment of RGFP966. (b) 134 proteins were downregulated after the stimulation of LPS and rescued by RGFP966.

**Figure 3:**
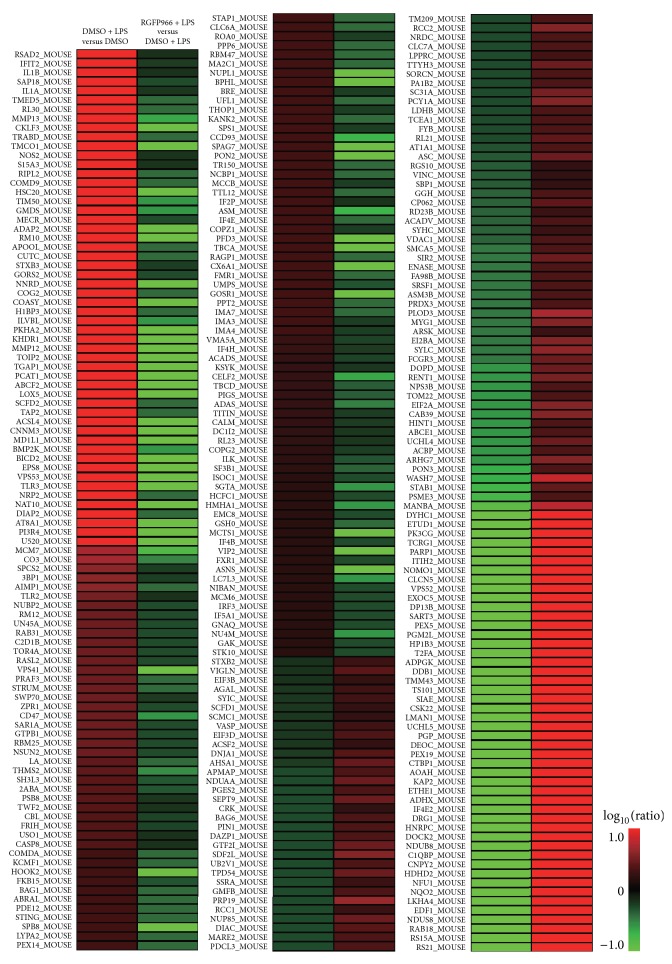
Heat map generated by MeV regarding proteins subjected to RGFP966 and LPS regulation. Experimental groups were presented on the horizontal axis and proteins were on the vertical axis. Colors were consistent with protein expression levels: red indicated upregulated ratio and green indicated downregulated ratio.

We identified > 1.5 fold, peptide > 1 as upregulated proteins and < 0.66 fold, peptide > 1 as downregulated proteins. The numbers of differently expressed proteins between two groups were listed in Table 2. Specifically, 168 of 444 (37.8%) LPS-induced proteins were significantly reduced with the treatment of RGFP966 (Figure 2(a)). Likewise, when analyzing proteins downregulated by LPS, the impact of RGFP966 was comparable, with 134 of 404 (33.2%) proteins being rescued by RGFP966 (Figure 2(b)). The heat map presented detailed information of the upregulated as well as downregulated proteins which were caused by LPS and simultaneously reversed by RGFP966 (Figure 3).

### 3.3. Gene Ontology Analysis

**Figure 4:**
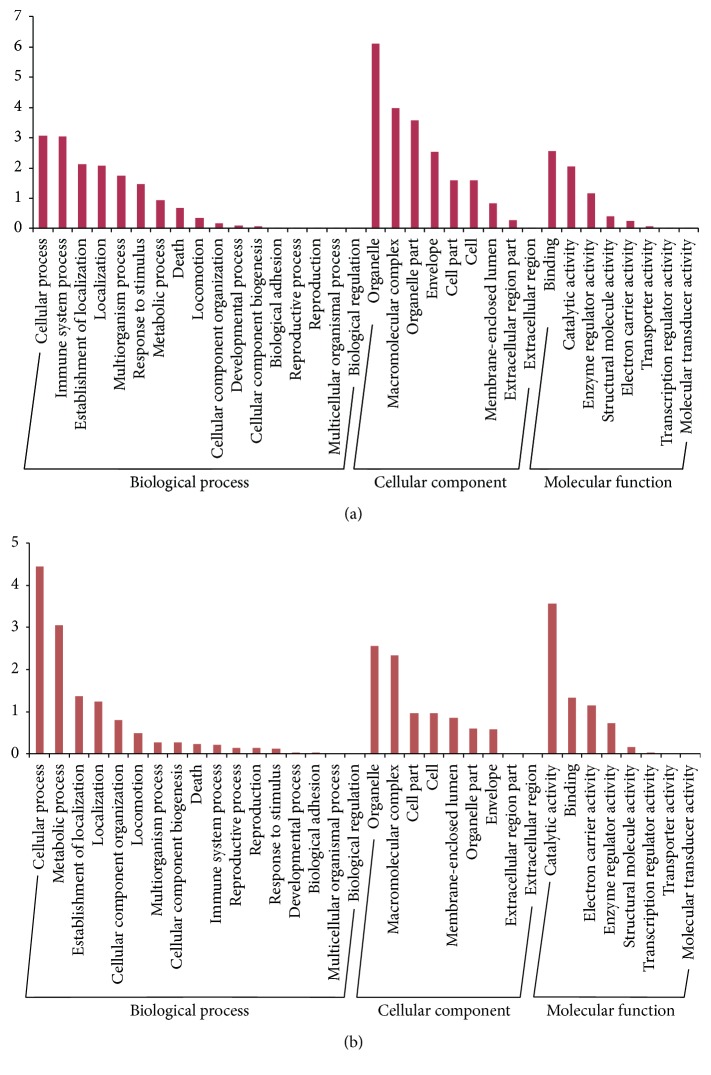
The GO analysis of differentially expressed proteins overlapped in Figure 2(a) (a) and Figure 2(b) (b). The GO analysis involved biological process, cellular component, and molecular function. The vertical axis values equaled −log⁡(*p*).

GO analysis was used to reveal the function of proteins in three aspects: cellular component, molecular function, and biological process. The 168 differentially expressed proteins overlapped in Figure 2(a) were mainly involved in biological process: cellular process, immune system process, and establishment of localization; cellular component: organelle, macromolecular complex, and organelle part; molecular function: binding, catalytic activity, and enzyme regulator activity (Figure 4(a)). However, the 134 differentially expressed proteins overlapped in Figure 2(b) were mainly involved in biological process: cellular process, metabolic process, and establishment of localization; cellular component: organelle, macromolecular complex and cell part; molecular function: catalytic activity, binding, and electron carrier activity (Figure 4(b)).

### 3.4. KEGG Pathway Analysis

**Figure 5:**
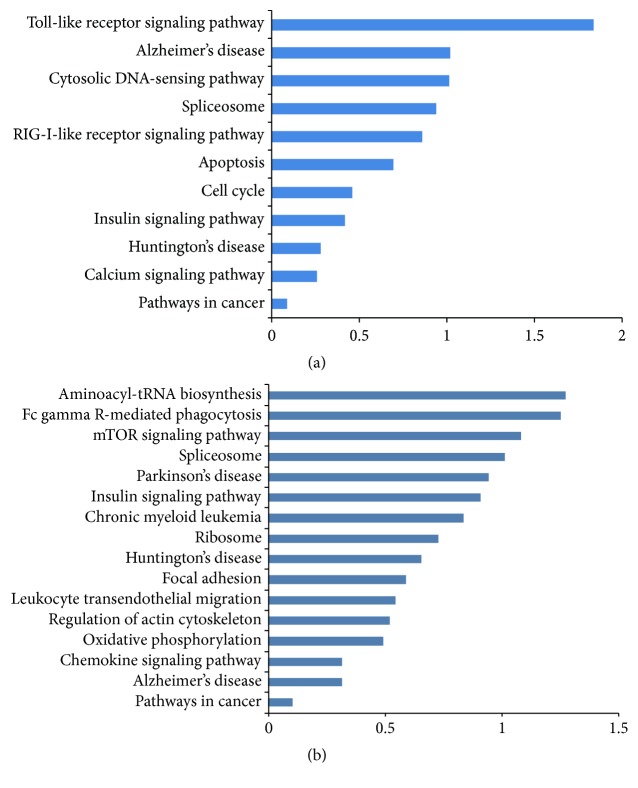
The KEGG pathway analysis of differentially expressed proteins overlapped in Figure 2(a) (a) and Figure 2(b) (b). The horizontal axis values equaled −log⁡(*p*).

To explore the potential signaling pathway through which HDAC3 specific inhibitor RGFP966 regulates inflammatory response, KEGG pathway analysis was performed. As shown in Figure 5(a), the pathway map depicted that the 168 differentially expressed proteins overlapped in Figure 2(a) were related to Toll-like receptor signaling pathway, Alzheimer's disease, cytosolic DNA-sensing pathway, spliceosome, RIG-I-like receptor signaling, apoptosis, cell cycle, insulin signaling pathway, Huntington's disease, calcium signaling pathway, and pathways in cancer. However, the 134 differentially expressed proteins overlapped in Figure 2(b) belonged to the following pathways: aminoacyl-tRNA biosynthesis, Fc gamma R-mediated phagocytosis, mTOR signaling pathway, spliceosome, Parkinson's disease, and so forth (Figure 5(b)).

### 3.5. mRNA Verifications of Discrepant Proteins between LPS Group and LPS + RGFP966 Group

**Figure 6:**
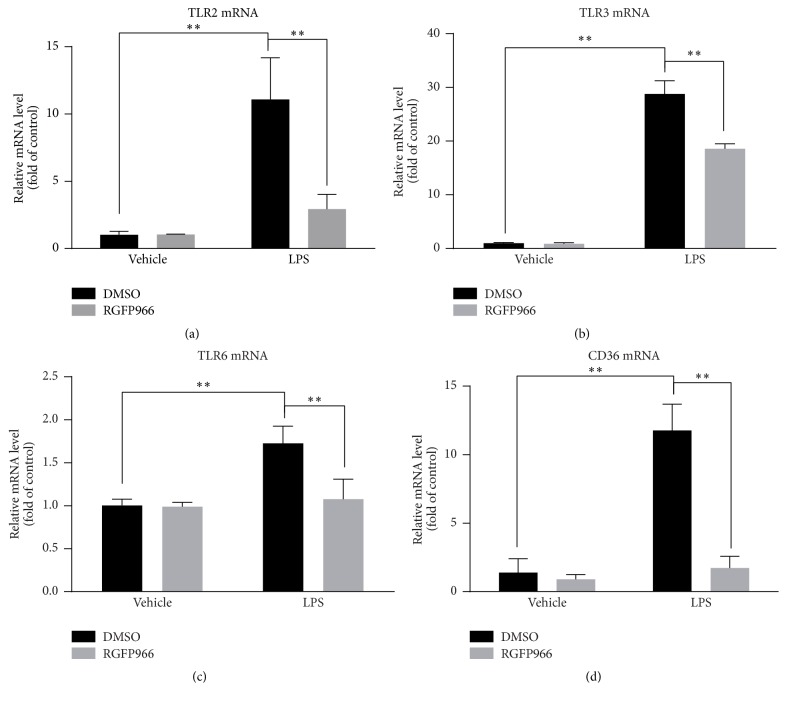
RT-PCR analysis of TLR2, TLR3, TLR6, and CD36 in four experimental groups. The mRNA levels of TLR2 (a), TLR3 (b), TLR6 (c), and CD36 (d) were significantly reduced by RGFP966 at the stimulation time of 6 hours. ^*∗∗*^*p* < 0.01. *N* = 3 repeats.

RT-PCR was performed to detect the mRNA levels of TLR2, TLR3, TLR6, CD36, and SYK at 6 hours after LPS stimulation. As expected, LPS stimulation potentiated profound mRNA increase of TLR2 (9.24 folds versus DMSO group), TLR3 (28.81 folds versus DMSO group), TLR6 (1.73 folds versus DMSO group), and CD36 (8.78 folds versus DMSO group). However, these augments were significantly decreased by RGFP966 with the decline amplitudes at 73.58% in TLR2 (*p* = 0.002), 35.51% in TLR3 (*p* < 0.001), 37.57% in TLR6 (*p* = 0.007), 85.32% in CD36 (*p* < 0.001), respectively (Figures 6(a)–6(d)). However, the mRNA expression level of SYK was not significantly altered (data not shown).

### 3.6. Protein Verifications of Discrepant Proteins between LPS Group and LPS + RGFP966 Group

**Figure 7:**
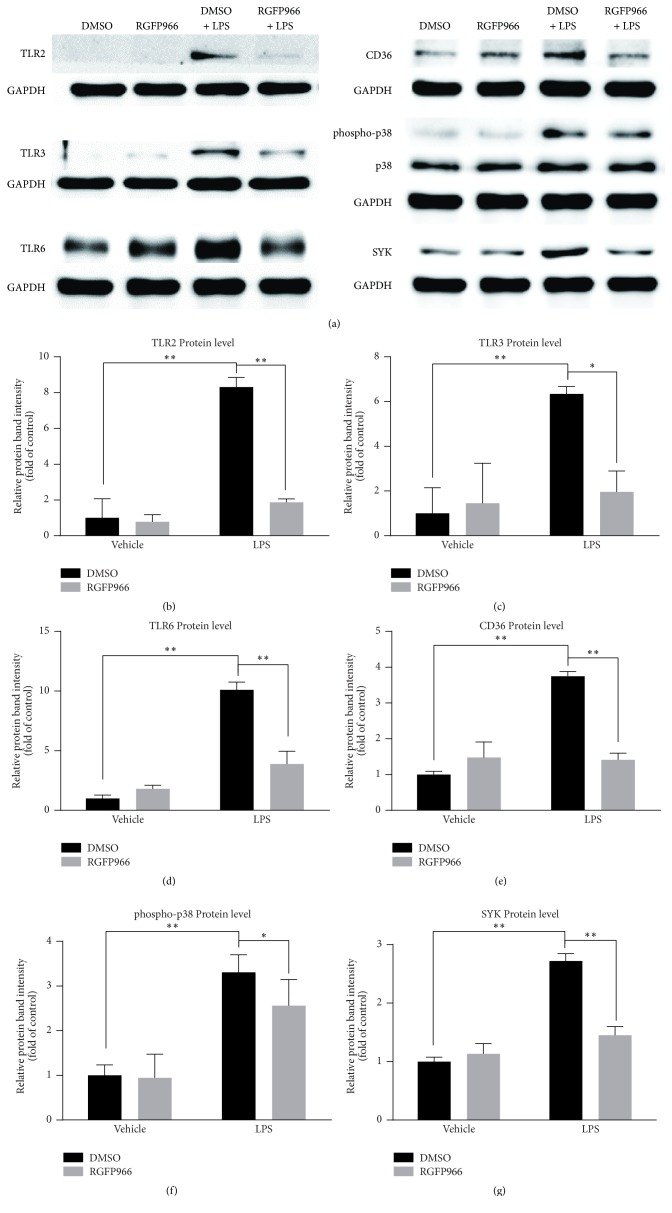
Western blot analysis of TLR2, TLR3, TLR6, MAPK p38, phospho-p38, SYK, and CD36 in four experimental groups (a). The protein levels of TLR2 (b), TLR6 (d), SYK (g), phospho-p38 (f), and CD36 (e) were significantly altered by RGFP966 after 12 hours' stimulation of LPS while TLR3 was at 24 hours. There was no significant difference of MAPK p38 protein expression among four groups. ^*∗*^*p* < 0.05, ^*∗∗*^*p* < 0.01. *N* = 3 repeats.

Then, we verified the changes of TLR2, TLR3, TLR6, CD36, and SYK at the protein level by western blotting (Figures 7(a)–7(g)). Consistent with the results of mRNA detection, the protein expression levels of TLR2 (8.31 ± 0.54 in DMSO + LPS versus 1.00 ± 1.07 in DMSO, *p* < 0.001; 1.87 ± 0.20 in RGFP966 + LPS versus 8.31 ± 0.54 in DMSO + LPS, *p* < 0.001), TLR6 (10.09 ± 0.66 in DMSO + LPS versus 1.00 ± 0.28 in DMSO, *p* < 0.001; 3.88 ± 1.07 in RGFP966 + LPS versus 10.09 ± 0.66 in DMSO + LPS, *p* < 0.001), CD36 (3.75 ± 0.13 in DMSO + LPS versus 1.00 ± 0.09 in DMSO, *p* < 0.001; 1.41 ± 0.18 in RGFP966 + LPS versus 3.75 ± 0.13 in DMSO + LPS, *p* < 0.001), and SYK (2.72 ± 0.13 in DMSO + LPS versus 1.00 ± 0.07 in DMSO, *p* < 0.001; 1.45 ± 0.15 in RGFP966 + LPS versus 2.72 ± 0.13 in DMSO + LPS, *p* < 0.001) were all significantly increased at the stimulation time of 12 hours and decreased with the treatment of RGFP966. In our study, though there was no significant change seen in total MAPK p38, the protein level of phospho-p38 (3.31 ± 0.40 in DMSO + LPS versus 1.00 ± 0.23 in DMSO, *p* < 0.001; 2.56 ± 0.58 in RGFP966 + LPS versus 3.31 ± 0.40 in DMSO + LPS, *p* = 0.021) showed a similar trend as the proteins mentioned above. TLR3 (6.34 ± 0.33 in DMSO + LPS versus 1.00 ± 1.15 in DMSO, *p* = 0.003; 1.96 ± 0.93 in RGFP966 + LPS versus 6.34 ± 0.33 in DMSO + LPS, *p* = 0.011), an exceptional protein, was significantly aggregated after LPS stimulation and reduced by RGFP966 at 24 hours.

### 3.7. RGFP966 Inhibited LPS-Induced Microglia Activation

**Figure 8:**
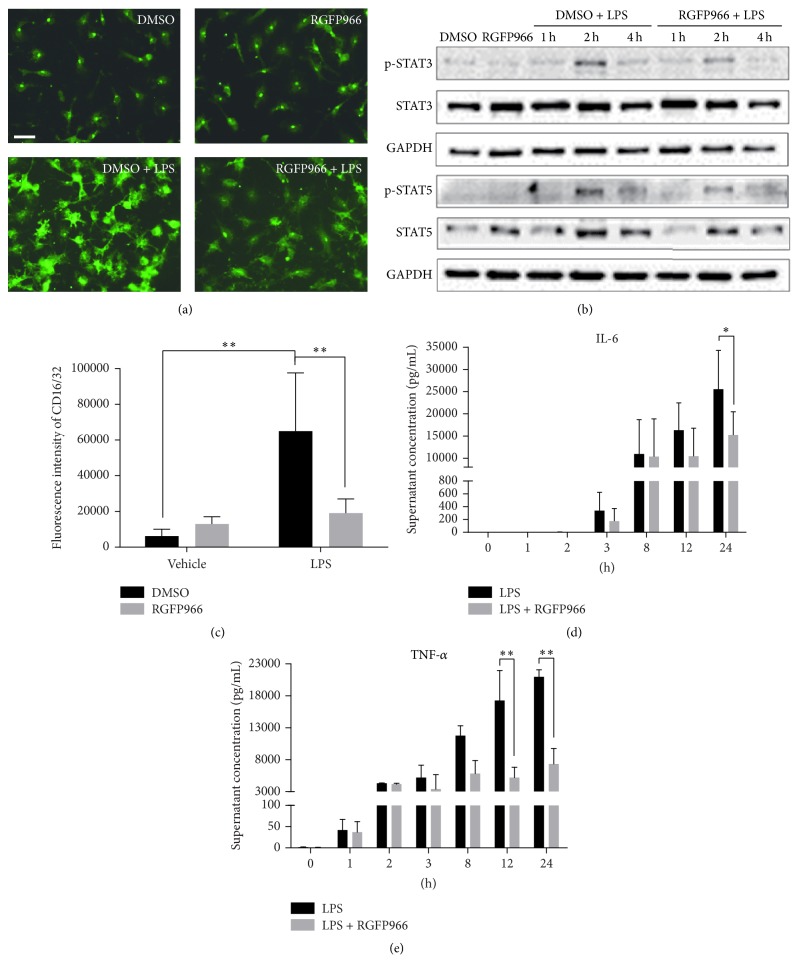
RGFP966 inhibited LPS-induced microglia activation. (a) Representative microphotographs of primary microglia cultures 24 hours after LPS and RGFP966 treatment. The cultures were stained with CD16/32, a marker of activated microglia. Scale bar = 10 *μ*m. (b) Expression and phosphorylation of STAT3 and STAT5 in microglia pretreated with RGFP966. (c) Quantitative analysis of fluorescence intensity of CD16/32. All the photographs used for quantitative analysis were taken under the same exposure time and data were expressed as fluorescence intensity per cell of randomly selected regions. ^*∗∗*^*p* < 0.01. *N* = 3 repeats. Supernatant concentration of IL-6 (d) and TNF-*α* (e) detected at various stimulation times. ^*∗*^*p* < 0.05, ^*∗∗*^*p* < 0.01. *N* = 3 repeats.

We further verified the effect of HDAC3 inhibition on functional microglia activation. We first stained primary cultured microglia with CD16/32, as shown in Figure 8(a), microglia demonstrated bipolar shape in intact status, while in LPS stimulation group, microglia enlarged and grew lots of branches. The fluorescence intensity of CD16/32 was significantly decreased in LPS + RGFP966-treated group (Figure 8(c)). We then detected classical inflammatory cytokines in the supernatants of cultured cells exposed to LPS for 1 h, 2 h, 3 h, 8 h, 12 h, and 24 h with or without RGFP966. We observed profound inhibition of IL-6 (Figure 8(d)) (24 h: 15230 ± 5213 pg/mL in RGFP966 + LPS versus 25538 ± 8741 pg/mL in DMSO + LPS, *p* = 0.012) and TNF-*α* (Figure 8(e)) (12 h: 5189 ± 1613 pg/mL in RGFP966 + LPS versus 17241 ± 4716 pg/mL in DMSO + LPS, *p* < 0.001; 24 h: 7332 ± 2436 pg/mL in RGFP966 + LPS versus 20977 ± 1091 pg/mL in DMSO + LPS, *p* < 0.001) secretion by RGFP966. Additionally, STAT3 and STAT5 were two fundamental signaling pathways governing inflammatory response in various neurological diseases [17]. In order to found out the mechanism responsible for its anti-inflammatory responses, we detected phosphorylation levels of STAT3 and STAT5. As shown in Figure 8(b), phosphorylation of STAT3 and STAT5 increased 2 h after LPS treatment and declined quickly 4 h after LPS. RGFP966 inhibited not only phosphorylation of STAT3 and STAT5, but also decreased STAT5 expression level, which may partially contribute to its anti-inflammatory response.

## 4. Discussion

In summary, we analyzed the differentially expressed proteins of LPS-stimulated primary microglia with the treatment of RGFP966 by proteomics. A total of 168 LPS-induced proteins were significantly reduced by RGFP966 in this study. The altered proteins mainly concentrated on the following categories: Toll-like receptor signaling pathway, Alzheimer's disease, cytosolic DNA-sensing pathway and functioned in cellular process, immune system process and organelle. For Alzheimer's disease, preliminary evidences demonstrated inhibition of HDAC3 enzyme in neurons prevented amyloid-beta oligomer-induced synaptic plasticity impairments [18] and enhanced memory process [15]. For DNA-sensing pathways, HDAC3 was thought to participate in IRF3/IFN-*β* signaling pathways [13]. In this study, we mainly analyzed the differentially expressed TLR relative proteins of LPS-stimulated primary microglia with the treatment of RGFP966. Toll-like receptor signaling pathway played an important role in the regulation of inflammation. In this regard, we selected TLR2, TLR3, TLR6, CD36, and SYK for further validation and found that they were all significantly upregulated after LPS stimulation and downregulated in the presence of RGFP966. Additionally, morphological and functional alterations of microglia exposure to LPS were also relieved by RGFP966. Our data provided a hint that HDAC3 inhibitor may be a potential therapeutic target combating microglia activation and inflammatory response in neurological diseases.

TLRs are a family of type I transmembrane receptors which share the multiple leucine-rich repeats (LRRs) domains in the extracellular space and the similar cytosolic domains of the interleukin-1 (IL-1) receptors [19]. TLRs are considered to recognize the distinct pathogen-associated molecular patterns (PAMPs) and endogenous damage-associated molecular patterns (DAMPs), which then drive a cascade of inflammatory signaling and converge at transcription factors nuclear factor-*κ*B (NF-*κ*B) [20], MAPK [21, 22], and JAK/STAT pathways [23]. In addition to TLR 2/3/6, TLR coreceptor CD36 and regulatory protein SYK were all decreased by RGFP966. Spleen tyrosine kinase (SYK), a nonreceptor tyrosine kinase, is typically regarded as a vital regulator in adaptive immunity [24]. It consists of two tandem SH2 domains and a C-terminal tyrosine kinase domain. SH2 domains selectively bind to the phosphorylated immunoreceptor tyrosine-based activation motifs (ITAMs) of immune receptors, such as MyD88, TRAF6, and TRIF, transmitting initiative signals to downstream pathways. SYK is found to be fundamental for Toll-like receptor signaling pathway and the inhibition of SYK suppresses the release of proinflammatory cytokines [25, 26]. In this study, we detected alterations of SYK in protein level but not mRNA level. We surmised that posttranscriptional modification may also contribute to HDAC3's functions. Preliminary evidences demonstrate that HDAC family played a role in protein degradation [27], while inadequate time points of mRNA detection could also be an explanation for this discrepancy. CD36 is a TLR coreceptor, playing a pivotal role in inflammatory responses initiated by TLRs [28]. Previous researches reported that CD36 contributed to the recognition of diacylglycerol ligands by forming CD36-CD14-TLR2-TLR6 complex; thus it controlled gram-positive bacterial infection. What is more, CD36 was found to be involved in the formation of TLR4-TLR6 heterodimers, which induced the production of proinflammatory cytokines, nitric oxide, and reactive oxygen species in response to endogenous ligands [29]. Overall, RGFP966 demonstrated profound inhibitory effects on TLR signaling pathway and proinflammatory cytokines including IL-6 and TNF-*α*.

**Figure 9:**
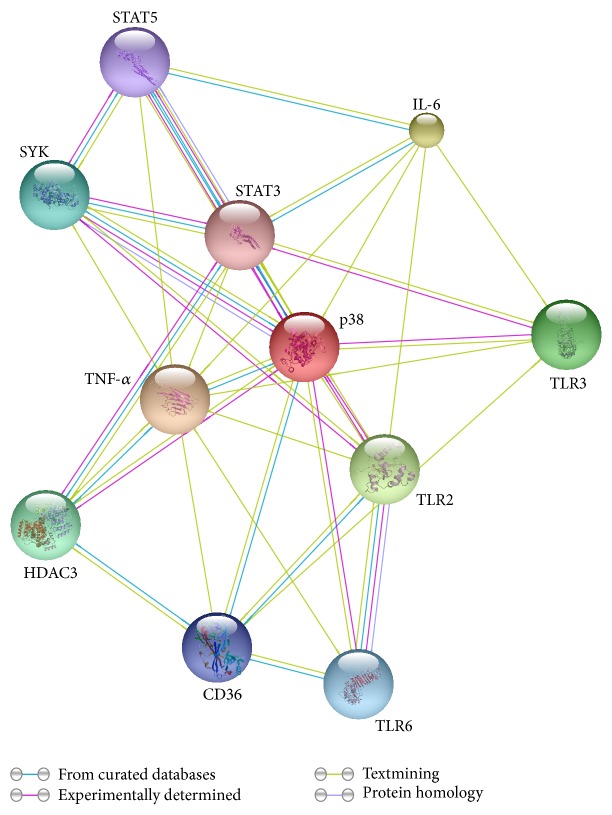
Protein-protein interaction network analysis. Each color of string represented interaction type, respectively.

HDAC inhibitors have been reported to interfere with the activation of the mitogen-activated protein kinases, STAT1, AP-1, or NF-*κ*B signal transduction pathways [30, 31]. Here, we observed transient STAT3 and STAT5 activation at 2 hours after LPS stimulation, which was ahead of the alteration of IL-6 and TNF-*α*. Exposed to inflammatory stimulation, STAT3/5 was phosphorylated by JAKs, leading to transcriptional activation. Activation of STATs elicited the expression of acute-phase proteins as well as a number of cytokines and chemokines, including IL-6 and TNF-*α* [17]. Our study was in keeping with previous study in HDAC3 knockout macrophage, which demonstrated that HDAC3 knockout impaired STAT1/STAT3/STAT5 pathways [13].  Figure 9 presented a diagram showing the protein and protein interactions among proteins. Briefly, initial change of STAT3/STAT5 or NF-*κ*B [5] caused by HDAC3i may further elicit later alterations of TLR signaling pathway and proinflammatory cytokines including IL-6 and TNF-*α*. The underlying mechanism for HDAC3 in the regulation of STAT3/STAT5 is unknown. Some evidences from Pan-HDAC inhibitors indicated that promoter-associated histone acetylation of SOCS1 and SOCS3 caused by HDACi may further downregulated JAK2/STAT3 signaling [32].

In conclusions, we identified TLR signaling pathway, microglia activation, and STAT3/5 pathway which were inhibited by RGFP966. Identification of these changes may provide valuable clues for the future applications of selective HDAC3 inhibitor in the clinic.

## Figures and Tables

**Table 1:** Summary of LC-MS/MS data.

Treatment	Total proteins
DMSO	1883
RGFP966	1967
DMSO + LPS	1806
RGFP966 + LPS	2024

**Table 2:** Summary of upregulated/downregulated proteins candidates.

	Upregulated protein candidates	Downregulated protein candidates
	> 1.5 fold, peptide > 1	< 0.66 fold, peptide > 1
RGFP966 versus DMSO	493	338
DMSO + LPS versus DMSO	444	404
RGFP966 + LPS versus DMSO + LPS	401	275
